# No evidence that rapid adaptation impedes biological control of an invasive plant

**DOI:** 10.1111/eva.13053

**Published:** 2020-08-18

**Authors:** M. Stastny, J.L. Russell‐Mercier, R.D. Sargent

**Affiliations:** ^1^ Department of Biology University of Ottawa Ottawa ON Canada

**Keywords:** enemy release, invasive species, population divergence, purple loosestrife, weed

## Abstract

Biological control is a popular tool for invasive species management, but its success in nature is difficult to predict. One risk is that invasive plants, which may have adapted to lower herbivore pressure in the introduced range, could rapidly evolve defences upon re‐association with their biocontrol agent(s). Previous studies have demonstrated that populations of the invasive plant purple loosestrife (*Lythrum salicaria*) exposed to biocontrol exhibit traits consistent with the rapid evolution of defence. However, to date, no one has tested this hypothesis under field‐natural levels of herbivory. Using seed from 17 populations of purple loosestrife growing in eastern Canada, that varied in their history of exposure to their biocontrol agent, the leaf beetle *Neogalerucella* spp., we transplanted 1,088 seedlings from 136 maternal families into a common garden under ambient herbivory. Over the following three and half years, we assessed plant performance in the face of biocontrol by measuring early‐season plant size, defoliation, flowering, and season‐end biomass. We discovered that a population history with biocontrol explained little variation in herbivory or plant performance, suggesting that adaptation is not hindering biocontrol effectiveness. Instead, plant size, subsequent defoliation, and spatio‐temporal variables were the main predictors of plant growth and flowering during the study. The high individual variability we observed in plant performance underscores that flexible strategies of allocation and phenology are important contributors to the persistence of invasive plants. Our findings suggest that plant adaptation to biocontrol is unlikely to be a strong impediment to biological control in this species, however, the high survival and variable defoliation of plants in our study also indicate that biocontrol alone is unlikely to result in significant population decline. We recommend that the application of multiple forms of control simultaneously (e.g. thinning plus biocontrol) could help to prevent the existence of refuges of large, reproductive individuals.

## 1 INTRODUCTION

Invasive species are a major source of biological and economic disruption. The predominant explanation for invasiveness, the “enemy release” hypothesis, posits that the rapid population growth and spread associated with invasion are largely due to a lack of (specialist) enemies in the introduced range (Callaway & Aschehoug, 2000; Elton, 1958; Keane & Crawley, 2002). Classical biological control (hereafter, “biocontrol”) aims to avoid some of the undesirable effects of other control strategies (e.g. herbicides) by re‐establishing the association of the invasive species (target) with its specialist natural enemy (biocontrol agent), such as an insect herbivore (Myers & Bazely, 2003). Enemy release thus provides the theoretical motivation for biocontrol: introducing a specialist enemy should establish population regulation of the invasive species. However, evidence supporting the enemy release hypothesis is mixed, and reduced herbivory does not appear to play a consistent role in the invasiveness of introduced plant species (e.g. Colautti, Ricciardi, Grigoriovich, & MacIsaac, 2004; Schultheis, Berardi, & Lau, 2015). Similarly, the success of biocontrol is difficult to predict; some programmes achieve adequate control of the target species, but others do not, and generalities that could guide future releases have proven elusive (Myers & Bazely, 2003).

Having lost most or all of the mutualists and antagonists with which they co‐evolved, invasive species encounter a novel set of ecological interactions and evolutionary pressures in the introduced range (Muller‐Scharer, Schaffner, & Steinger, 2004). This premise is the basis for the “evolution of increased competitive ability or EICA” hypothesis (Blossey & Notzold, 1995), which postulates that an invasive plant's competitive advantage in its new environment is at least partially a product of postintroduction evolution: genotypes that invest less into defence and more into growth are predicted to have higher fitness. Although support for EICA has been inconsistent (Felker‐Quinn, Schweitzer, & Bailey, 2013; Rotter & Holeski, 2018), one important implication of EICA is that, upon re‐association with natural enemies (either accidental or through biocontrol), the invasive plant may re‐evolve higher investment in defence (Franks, Pratt, Dray, & Simms, 2008), as better defended genotypes have a fitness advantage under herbivory. Because rapid evolution of defence has the potential to weaken the effectiveness of biocontrol programmes (Muller‐Scharer et al., 2004), drive biocontrol agents to alternative hosts (Stenberg, Hamback, & Ericson, 2008), and/or enhance the noxious properties associated with defence (Zangerl & Berenbaum, 2005), understanding the likelihood and magnitude of this outcome is an important goal of invasion biology.

Plant defence traits that have evolved in response to selection imposed by herbivores are typically classified as either resistance or tolerance. Resistance traits are those that involve physical or chemical defences to deter or reduce herbivory (Berenbaum, Zangerl, & Nitao, 1991). Some resistance traits function primarily against specialist herbivores and may become redundant in their absence, while others may target generalists, potentially conferring advantage in the introduced range (Muller‐Scharer et al., 2004). Tolerance traits are those that help mitigate the impacts of herbivory on plant fitness, such as the ability to regrow or reallocate following damage (Strauss & Agrawal, 1999), or to alter phenology (Benning, Eckhart, Geber, & Moeller, 2019; Pilson, 2000), including the timing of flowering (Austen, Rowe, Stinchcombe, & Forrest, 2017; Pilson, 2000) to avoid the heaviest costs of herbivory. Most plant species probably rely on both strategies, and variability in their mode of action, costs, and effectiveness against different herbivores complicates our ability to predict changes in plant defensive phenotypes in novel environments (Carmona & Fornoni, 2013).

While evolutionary change in plant defence in response to herbivory is well‐documented (Agrawal, Hastings, Johnson, Maron, & Salminen, 2012; Ågren, Hellström, Toräng, & Ehrlén, 2013; Benkman, Smith, Maier, Hansen, & Talluto, 2012; Uesugi, Connallon, Kessler, & Monro, 2017), few studies have examined whether an invasive plant's defence strategies evolve following re‐association with a specialist enemy. In one well‐known example, Zangerl and Berenbaum, (2005) reported that invasive wild parsnip plants collected during their early establishment period exhibited markedly lower levels of defensive furanocoumarins than plants collected after the accidental introduction of a specialist herbivore, the parsnip webworm. Their findings strongly implicate the evolution of increased herbivore resistance following re‐association. Other studies have found conflicting evidence of adaptation to biocontrol. In a study of adaptation of tansy ragwort, (*Jacobea vulgaris*) to its introduced biocontrol agent, the herbivorous Chrysomelid beetle *Longitarsus jacobaeae*, Rapo, Müller‐Schärer, Vrieling, and Schaffner, (2010) reported that New Zealand populations exposed to biocontrol exhibited lower levels of alkaloids compared to those never exposed, while in North America the pattern was reversed. On the other hand, Franks et al. (2008) found no evidence of evolutionary change in defence in Floridian populations of the invasive plant *Melaleuca quinquenervia* following the introduction of two herbivorous biocontrol agents.

Evolution of increased resistance or tolerance in response to biocontrol would require sufficiently strong selection by the herbivore and genetic variation in defence traits. Biocontrol agents are chosen specifically for their significant impacts on growth and reproduction of the invasive species, necessary for population regulation (Myers & Bazely, 2003); therefore, these herbivores should impose genotype sorting and strong selection on plant defences, especially in the period immediately following their release (Muller‐Scharer et al., 2004). And while introduced populations are often genetically less diverse than those in the native range, due to processes such as bottleneck effect associated with the introduction (Dlugosch & Parker, 2008), a number of studies have shown rapid adaptation in the introduced range (Rapo et al., 2010; Uesugi et al., 2017; Zangerl & Berenbaum, 2005). One well‐known example is *Lythrum salicaria* (purple loosestrife), a common Eurasian invader of wetlands in North America. This species possesses an impressive ability to adapt rapidly to the novel conditions encountered in its introduced range, including clear evidence of earlier flowering phenology along its northward expansion (Colautti & Barrett, 2013). In the early 1990s, a biocontrol programme was initiated in the Canadian province of Ontario by releasing two species of specialist Chrysomelid leaf‐feeding beetles *Neogalerucella calmariensis* and *N. pusilla*, (hereafter, *Neogalerucella* spp.) (Blossey, 1995). In spite of widespread establishment of the biocontrol, the success of the programme has been equivocal, with many *L. salicaria* populations showing little or no evidence of reduction in plant height, density or propensity to flower in the years since release (Grevstad, 2006; Hovick & Carson, 2015; St. Louis, Stastny, & Sargent, 2020). This outcome raises the question of whether the efficacy of biocontrol may be compromised by the adaptation of the target invader following the agent's release.

In our study region of eastern Ontario/western Quebec, *L. salicaria* populations vary in their exposure to and history of biocontrol, ranging from sites of the original biocontrol release to sites with no previous exposure. This context of putatively different regimes of natural selection by the specialist herbivore offers an opportunity to examine the possibility and ecological consequences of rapid adaptation in resistance or tolerance to biocontrol. Two separate studies have demonstrated that plants from *L. salicaria* populations with a longer period of exposure to *Neogalerucella* spp. appear to be better defended against this specialist than plants from populations without a history of re‐association (Quiram, 2013; Stastny & Sargent, 2017). However, previous studies either examined plant defence traits in situ, making it difficult to disentangle population history from other site‐specific factors, or in highly controlled greenhouse/field situations that differ significantly from natural ecosystems. Herbivory is difficult to replicate under highly controlled, low‐stress environments that may magnify the expression of genetic differences (Johnson, Dinnage, Zhou, & Hunter, 2008), making the existing results difficult to extrapolate to the natural settings where predicting biocontrol success is most relevant.

The goal of this multi‐year study was to test whether, under field conditions, a population history with biocontrol would predict plant performance in the face of herbivory. Over three and half years in a wetland common garden with a robust local population of the biocontrol agent *Neogalerucella* spp., we grew over 1,000 plants from 17 *L. salicaria* populations that varied with respect to their length of prior exposure to *Neogalerucella* spp. (between zero and ~ 20 years) under ambient herbivory and plant competition. Specifically, we asked: (1) Do populations with a history of exposure to biocontrol experience less damage in a natural setting? and (2) How do field levels of herbivory, and its variability over space and time, affect plant performance?

## 2 MATERIALS AND METHODS

### 2.1 Study species

A wetland invasive of European origin, *Lythrum salicaria* L. (Lythraceae) is a tristylous, perennial herb that has undergone a relatively rapid expansion throughout North America, following multiple introductions for horticulture during the 19th and 20th centuries. Flowers are self‐incompatible and are mainly pollinated by bees. Each flowering stem can produce up to ~100,000 seeds (Montague, Barrett, & Eckert, 2008). Although *L. salicaria's* North American lifespan is not well‐documented, genets persist for multiple years even under severe herbivory, and the same plant can regrow from its rootstock after not producing any above‐ground biomass the previous year (Thompson, Stuckey, & Thompson, 1987). As such, any differences between populations with different exposure to biocontrol would likely be due largely to genotype sorting, although we note that previous studies have found that *L. salicaria* has rapidly (i.e. within < 50 years) evolved significant local adaptation to climatic variation associated with latitude (Colautti, Agren, & Anderson, 2017). In the early 1990s, in response to concerns about the impact of *L*. s*alicaria* on the integrity of wetland ecosystems (Anderson, 1995), several North American governments, including the Canadian province of Ontario, introduced a pair of specialist leaf‐eating beetles from the species’ European range for the purpose of biocontrol. In our study region, *Neogalerucella* spp. typically complete two generations in a single growing season: mid‐ to late‐spring larval feeding can strongly impact growth, plant architecture and reproduction, while the subsequent, mid‐summer generation also feeds on developing inflorescences (Dech & Nosko, 2002), directly impacting fitness. *Lythrum salicaria* plants exposed to feeding by *Neogalerucella* spp. exhibit a defensive strategy most consistent with tolerance, including compensatory growth and delayed phenology (Quiram, 2013; Thomsen & Sargent, 2017). Follow‐up studies on the success of North American biological control programmes have reported a mixture of outcomes, with some exposed *L. salicaria* populations exhibiting declines in plant size, flowering and abundance, but not others (Denoth & Myers, 2005; Grevstad, 2006; Hovick & Carson, 2015; St. Louis, 2020).

### 2.2 Plant material

In 2012, *L. salicaria* seeds were collected from flowering individuals from 17 populations throughout a climatically uniform region of eastern Ontario and western Quebec, Canada, spanning ~1 degree of latitude near Ottawa (see Stastny et al. (2017) for details). Six of the populations were selected from the original release sites of *Neogalerucella* spp. in eastern Ontario (Corrigan, 2006; St. Louis et al., 2020) in the 1990s (hereafter, “release”). Another six populations were identified as having been colonized by *Neogalerucella* spp. during its subsequent spread (hereafter, “recent”). The final five populations had not experienced any beetle herbivory (hereafter, “naïve”), as repeated field surveys of the sites failed to detect any beetles or damage (St. Louis et al., 2020; Stastny & Sargent, 2017). As much as possible, our selection of populations aimed to avoid any systematic bias in site or population characteristics with respect to the history of biocontrol, disturbance or colonization by *L. salicaria*. At each site, we collected seeds from approximately 40 flowering plants, spaced at least 5 m apart; these represent half‐sib maternal lines (hereafter, families).

In late March 2013, we selected eight families per population that captured the phenotypic mean and variance of each population as assessed previously in the greenhouse; these same families and seed material were reported on in a previous experiment (Stastny & Sargent, 2017). The seeds were bulk‐germinated under greenhouse conditions on moist soil (Metro‐Mix, Sun Gro Horticulture) in narrow plastic “conetainers” (66 ml; Stuewe & Sons Inc.) with added side perforations to encourage lateral root growth, and randomized in holding racks that were then placed into bottom‐watering trays (Stuewe & Sons Inc.). The seedlings were thinned randomly until only a single plant remained in each conetainer to yield a total of 1,088 plants (17 populations x 8 families x 8 replicates), grown at 20: 13^0^C and at 16:8 hr light: dark photoperiod. Weekly, we rotated the racks among the watering trays to minimize positional effects. In mid‐May, after 6–7 weeks, we transferred the plants under shade cloth to a rooftop garden for approximately two weeks to allow acclimation to outdoor light levels and temperatures. In early June 2013, just before transplanting into the field, we measured plant height after 8–9 weeks of growth (hereafter, “initial size”); this metric of early vigour is strongly correlated with biomass in young plants before branching (M. Stastny, unpublished data).

### 2.3 Common garden

The plants were then transplanted into an open wetland in an old field near Ashton, Ontario (45.193°N, 76.026°W), located within our sampling region. The site has a robust *L. salicaria* population (>30,000 genets; pers. obs.), and relatively high densities of *Neogalerucella* spp., as well as two less abundant, introduced specialist herbivores: the florivorous weevil *Nanophyes marmoratus* and the aphid *Myzus lythri*.

In a randomized block design, we transplanted our plants into four plots (blocks), each containing 272 plants (2 replicates of each family per plot, from 8 families in each of the 17 populations, i.e. 136 families), in a regular grid with 1 m spacing. The plants were planted directly in their perforated conetainers, to help distinguish them from the existing *L. salicaria* plants and to facilitate their eventual removal, without hindering their growth. The surrounding vegetation was trimmed twice in year 1 to aid their establishment, and the plants were fertilized with a 10–20–10 (N, P, K) solution mid‐season. At the request of the landowners, we did not use insecticides to exclude herbivory. It also proved impractical to add insect exclusion cages for logistical reasons in addition to their confounding effects on plant growth. We therefore allowed the plants to grow for the subsequent three years under field conditions including competition from other plants, and under the natural levels of herbivory by *L. salicaria's* main biocontrol agent, *Neogalerucella* spp.

### 2.4 Data collection

Twice each growing season, and always by the same observer (M. Stastny), we measured herbivory by *Neogalerucella* spp. by visually estimating the percentage (to the closest 10%) of the total leaf area of each plant consumed by the insect (hereafter, % damage; see Johnson, Bertrand, and Turcotte, (2016) for methodological details). The first damage estimate, coinciding with the end (pupation) of the first larval generation of *Neogalerucella* spp., was taken each year in late June or early July. These data represent the combined herbivory by the overwintering adult beetles and their larval‐stage progeny, with the latter inflicting most of the damage. The second damage estimate, in mid‐August, coincided with the end of the second larval generation. This late‐season bout of defoliation, combining both larval and adult beetle herbivory, was considerably lighter than the first (due to lower insect densities and larger plant biomass) but showed similar patterns; therefore, in the rest of the paper we focus on the early‐season damage.

At the end of each complete growing season (i.e. years 1 through 3), above‐ground biomass was harvested once most of the foliage had senesced, dried for 48 hr at 50^0^C and weighed. These measurements (hereafter, “season‐end biomass”) represent for each individual the combined outcome of above‐ground allocation and herbivory, including plant compensation for damage (see below).

Every spring, the plants then regrew from their below‐ground parts, typically as multiple ramets, which is characteristic of *L. salicaria's* habit (Montague et al., 2008). In years 2 and 3, immediately prior to the first larval generation of *Neogalerucella* spp. (late May to early June), we measured the height of all stems (ramets) for each plant and used their sum as an estimate of early‐season size; this metric is strongly correlated with above‐ground biomass in the spring (M. Stastny, unpublished data). In the final (4th) year of the experiment, prior to the termination of the common garden, we harvested all plants in late May to obtain their early‐season above‐ground biomass and then safely dispose of them. Therefore, our measurements of early‐season plant size in each of the years 2 through 4 represent an estimate of above‐ground allocation and phenology of regrowth prior to any significant damage by the biocontrol agent. In the analyses (see below), we use initial size at transplanting (year 1) as a covariate along with the early‐season size in subsequent seasons. However, we only use early‐season size as a response variable (see below), as it has a distinct interpretation for natural patterns of plant regrowth and phenology after overwintering in situ, whereas initial size is largely the outcome of greenhouse conditions prior to transplanting. We standardized (i.e. scaled and centred) early‐season size within each of the three years (2, 3 and 4) prior to analysis, allowing us to directly compare these metrics across study years.

The majority of plants (>95%) survived until we ended the experiment in the late spring of year 4, although only a few flowered in the first two years. A proportion of plants that survived were recorded as “missing” during one or more surveys, if a) they did not initiate regrowth until after the early‐season census (*N* = 54 and 289 plants missing but surviving in years 2 and year 3, respectively), or b) only below‐ground biomass remained after particularly severe herbivory and the plant did not reappear until the following year (*N* = 14 and 185 plants in year 2 and year 3, respectively). Starting in mid‐summer of year 3, we surveyed all the plants every few days to record the date of the formation of inflorescence and the opening of the first flowers.

### 2.5 Statistical analyses

To test whether the history of biocontrol predicted patterns of herbivory (i.e. defoliation) across years 1 through 3, we used a generalized linear mixed‐effects model from the negative binomial family (glmer.nb with logit link function) that best accommodated the skewed distribution of the defoliation data. The model included plant size at the onset of herbivory (initial size at transplanting in year 1, and early‐season regrowth in years 2 and 3) as a covariate, and the fixed factors of population history of biocontrol (naïve, recent or release), year (1 – 3) and plot (1 – 4). Population and half‐sib family were included, along with individual plant ID, as nested random factors to account for repeated measurements over time, allowing us to examine whether there were significant inter‐population or inter‐family differences in the various response variables. We also examined covariation in the levels of herbivory between years, as an indication of genotypic differences in resistance to *Neogalerucella* spp., by calculating mean defoliation for each of the 136 half‐sib families for each year (using up to 8 replicates per family); these family means were then used in Pearson's correlation tests.

We tested whether the history of biocontrol explained variation in emergent (early‐season) plant size across years 2–4 using a linear mixed‐effects model (function lmer) with the same basic structure as above but without a covariate, on a log‐transformed, scaled response variable (early‐season size). In addition, we repeated this analysis by separately including the following covariates corresponding to the preceding year (i.e. years 1, 2 and 3): the defoliation each plant had suffered, or the season‐end biomass it had reached. These two analyses thus examined whether damage or plant performance in a previous year impacted early plant growth the following spring (i.e. years 2, 3 and 4), and whether this relationship was contingent on the history of biocontrol (i.e. yielded a significant interaction).

We examined the effects of population history and *Neogalerucella* spp. herbivory on plant biomass across years 1 to 3 in a linear mixed‐effects model (function lmer) with season‐end biomass as a log‐transformed response variable and scaled early‐season plant size and defoliation as covariates. The model also included an interaction term between defoliation and history of biocontrol, as an indirect test of population differences in tolerance to herbivory (i.e. to compare the slopes of regression lines between defoliation and season‐end biomass); all of the other fixed and random factors in the model were as specified above.

A generalized linear mixed model (logistic regression, function glmer) was used to assess the effect of a plant's population history, early‐season plant size and damage on reproduction (i.e. whether it produced flower buds and/or flowers) and its phenology in year 3.

We confirmed that the residuals from all the models above fit the assumptions of linear (or generalized linear, where appropriate) mixed models and checked variance inflation factors (function vif) for the models involving covariates; no elevated VIF were found for any model we report on below.

All analyses were conducted using R statistical software (version 3.6.1, R Core Team 2019), packages lme4 and car.

## 3 RESULTS

### 3.1 Patterns of herbivory

**Figure 1:**
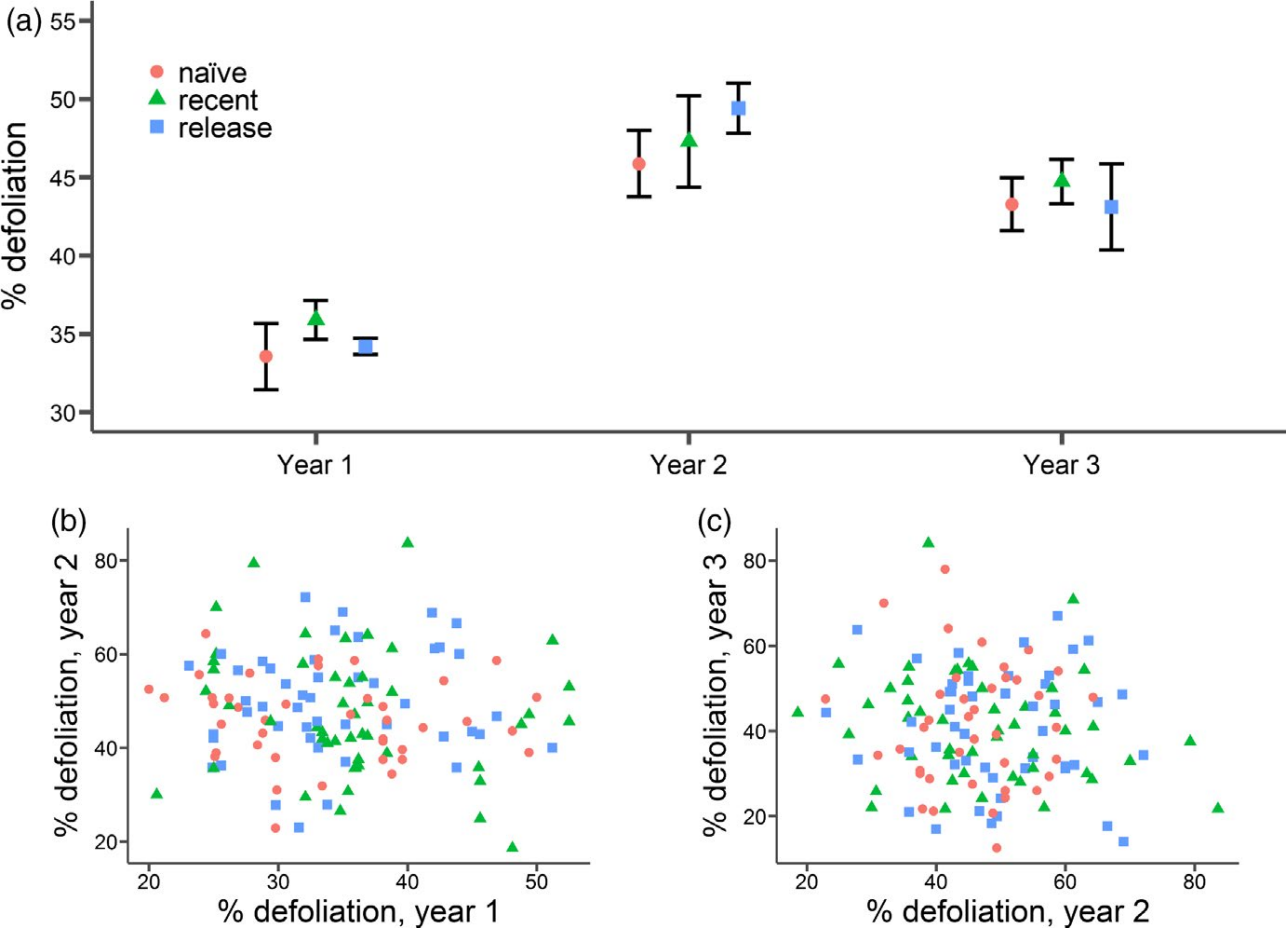
(a) Means (± SE) of per cent defoliation of *Lythrum salicaria* by the early‐summer generation of *Neogalerucella* spp. leaf beetles across years 1, 2 and 3 for the three sets of populations differing in the history of biocontrol (naïve = no prior exposure; recent = secondary colonization, ~5–15 years; release = sites of biocontrol release, ~20 years ago). Bottom panels: scatterplots of 136 half‐sib family means, showing the correlation between defoliation (b) in year 1 versus year 2 and (c) in year 2 versus year 3. The symbols and colours indicate the family's history of biocontrol, as above

Whether considered collectively (repeated measures model) or separately for each year (not shown), a history of biocontrol did not predict a plant's resistance to defoliation by *Neogalerucella* spp. (Figure 1a, Table 1, F2, 1,085 = 0.176, *p* = .816), with most populations showing similar levels of defoliation (Figure S1). The level of damage was highly variable among individual plants in the field, ranging from 0% to complete defoliation (100%) and characterized by skewed distributions (median early‐season damage in each of the three years: 30%, 35% and 35%, respectively). Notably, while herbivory varied significantly across years, a plant's level of defoliation in one year was not a predictor of defoliation in another year, whether at the level of individuals (Figure S2), or family means (Figure 1b; year 1 versus year 2: Pearson's *r* = −0.0610, *p* = .482; Figure 1c; year 2 versus year 3: Pearson's *r* = −0.110, *p* = .215). Furthermore, although damage varied with early‐season plant size, this relationship differed from year to year, ranging from negative to positive (Figure S3). We found that an individual's size at the onset of *Neogalerucella* spp. herbivory (i.e. size at the time of transplant in year 1, or early‐season size in years 2 and 3) was a significant predictor of both defoliation and season‐end biomass (Table 1). Neither population nor family were significant terms in any of the models. Both year and plot (block) explained a significant amount of variation in defoliation (Table 1), highlighting the substantial variability in herbivory across space and time, which is a known characteristic of this system (Denoth & Myers, 2005).

**Table 1:** Linear mixed model of impact of common garden factors on a plant's defoliation, early‐season size, and season‐end biomass

Response Variable	Model	Fixed Factors	F	*df*	*p*‐value
Defoliation (years 1–3)	glmer.nb	Population History	0.708	2	.472
		Plot (Block)	6.65	3	.00019
		Year	22.9	2	<.0001
		Early‐season Size	157.6	1	<.0001
Early‐season size (years 2–4)	lmer	Population History	0.465	2	.552
		Plot (Block)	9.54	3	<.0001
		Year	25.7	2	<.0001
Season‐end biomass (years 1–3)	lmer	Population History	1.60	2	.248
		Plot (Block)	14.8	3	<.0001
		Year	195.6	2	<.0001
		Early‐season Size	252.0	1	<.0001
		Defoliation	337.9	1	<.0001
		Defoliation x History	0.528	2	.611

### 3.2 Plant growth

**Figure 2:**
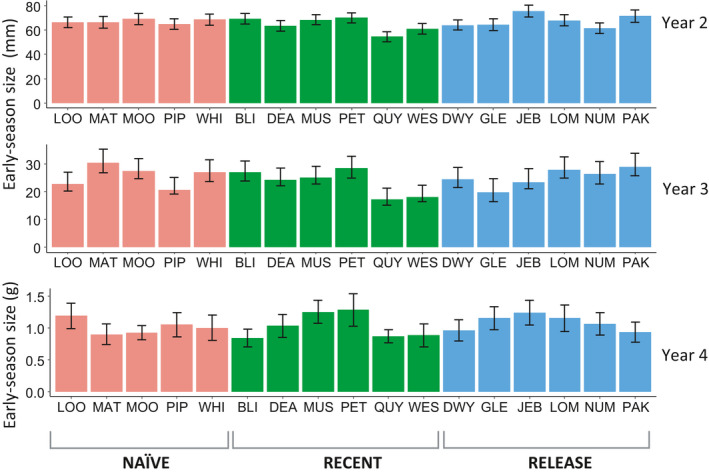
Variation among 17 populations of *Lythrum salicaria* differing in the history of exposure to biocontrol, showing means (±SE) of early‐season plant size following overwintering and prior to the onset of herbivory by the early‐summer generation of the specialist *Neogalerucella* spp. leaf beetles. Plant size in years 2 and 3 was measured as the sum of all stem heights (see text for details), while dry above‐ground biomass was measured in year 4

In spite of the initial differences in among‐family variation in vigour under greenhouse conditions (likelihood ratio test, family effect: *χ^2^* ^^= 4.10, *p* = .0430), plants from populations in the naïve, recent and release categories had attained a similar initial size by the time of transplanting into the common garden (F_2, 1,085_ = 0.176, *p* = .816). Likewise, under field conditions and preceding season herbivory, early‐season size did not differ with respect to the population's history of biocontrol (Figure 2), although it varied strongly over time and space (year and plot effects, Table 1). While performance in the greenhouse (i.e. year 1) did not predict plant size in the early season of year 2, early‐season size was correlated across subsequent years (i.e. year 2 versus year 3, and year 3 versus year 4), both at the level of individual plants (Figure S4a) and family means (Figure S4b). In other words, some individuals and families consistently allocated more to vegetative growth in the spring than others and/or differed in the timing of regrowth. Variation in herbivory and plant performance resonated into subsequent years: defoliation experienced by the early summer and season‐end biomass reached by fall of the preceding year were both highly significant predictors of early‐season plant size the following year (Table 1). However, while the relationship with defoliation did not vary with the history of biocontrol (Table 1), the positive covariance between a plant's season‐end biomass and subsequent early‐season size was slightly steeper for plants from the release populations compared to those in naïve populations (biomass x population history interaction: *F*_1,2_ = 5.70, *p* = .00380; Table 1).

**Figure 3:**
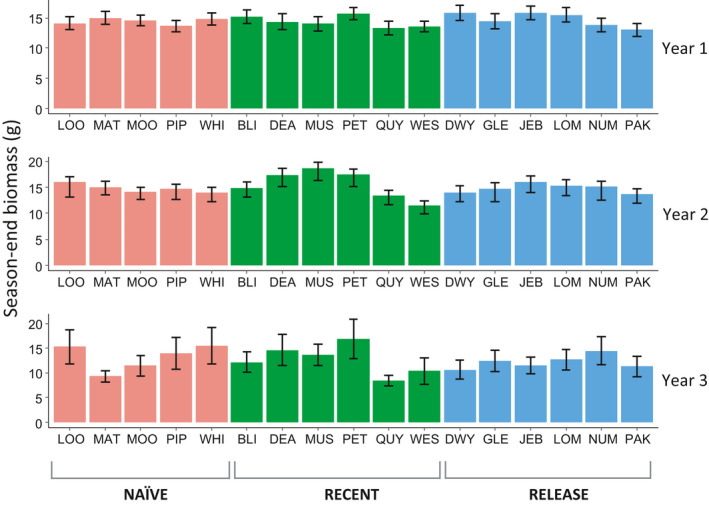
Variation among 17 populations of *Lythrum salicaria* differing in the history of exposure to biocontrol, showing means (±SE) of season‐end above‐ground biomass after a season of herbivory by the specialist *Neogalerucella* spp. leaf beetles

Both plant size early in the season and the level of defoliation sustained in early summer were significant predictors of biomass by the end of the growing season in each year (Table 1). However, in spite of the large range of variation in individual plant performance, we found no differences in season‐end biomass with respect to the history of biocontrol across the three years or for any specific year with respect to the history of biocontrol (Table 1, Figure 3). The effect of a plant's size at the onset of herbivory (i.e. size at transplanting in year 1, or early‐season size in years 2 and 3) on its season‐end biomass was positive—a one unit increase in initial plant size was roughly equivalent to a one unit increase in season‐end biomass. The effect of herbivore damage was similar; a one unit increase in % damage was associated with a one unit decline in season‐end plant biomass, irrespective of the history of biocontrol (i.e. a defoliation x history interaction was not significant; Table 1). As with *Neogalerucella* spp. herbivory and early‐season plant size, time and space strongly predicted season‐end biomass (year and plot effects, Table 1); plant biomass accumulation in years 2 and 3 was significantly lower than in year 1 (Figure 3), in spite of lower herbivory overall.

**Figure 4:**
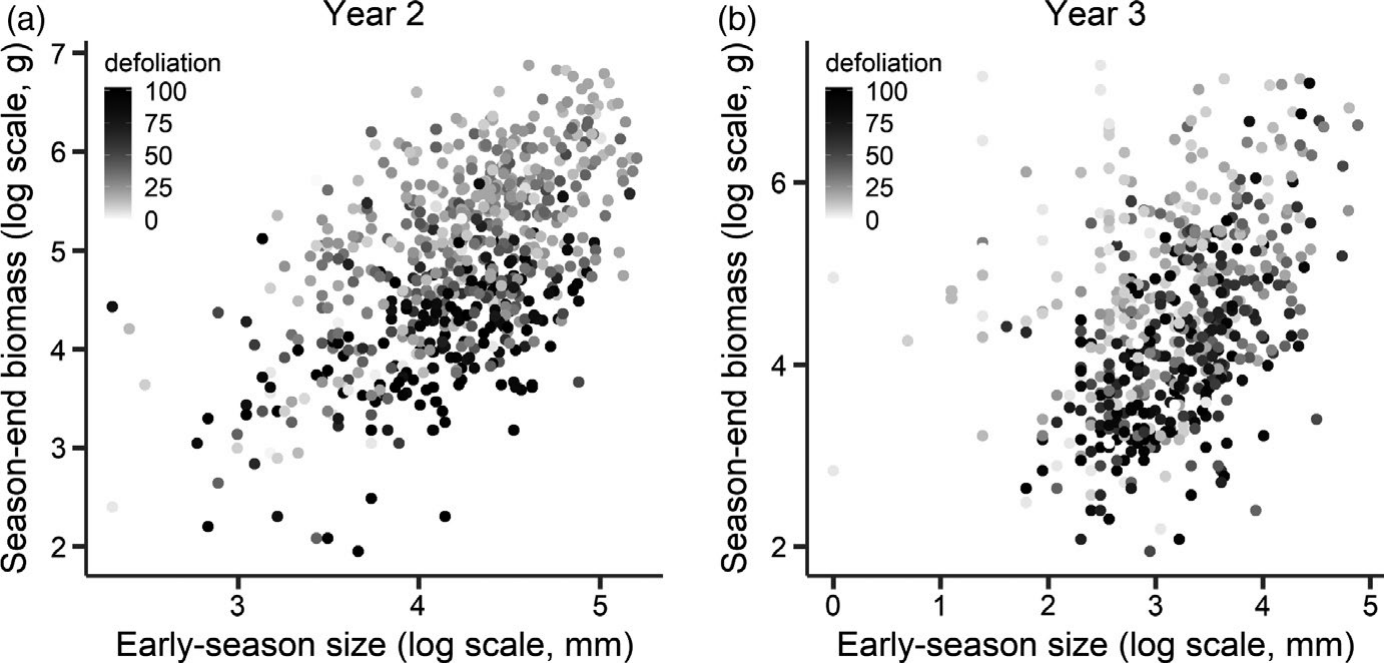
Scatterplots of individual *Lythrum salicaria* plants (a) in year 2 and (b) year 3 of the common garden experiment, showing early‐season plant size (x‐axis, log‐scale), season‐end biomass (y‐axis, log‐scale), and the degree of defoliation by early‐summer generation of the specialist *Neogalerucella* spp. leaf beetles (see legend for grey scale: darker shades indicate higher defoliation)

We did not detect significant variation among populations or families for any of the three main metrics above, probably because it was swamped by the considerable variation among replicates and across years. Therefore, we then examined how, at the level of individual plants, early‐season vigour prior to herbivory and the degree of *Neogalerucella* spp. damage jointly explain the variation in plant performance in years 2 and 3. In both year 2 and year 3, individuals that achieved the highest biomass by the end of the growing season tended to be the most vigorous (or advanced in phenology) early in the season prior to the first bout of herbivory and then suffered relatively lower defoliation (Figures 4a and b, respectively). Conversely, those individuals that tended to underperform in terms of season‐end biomass tended to exhibit lower to intermediate vigour (or delayed phenology) early in the season and then suffered heavier defoliation. However, by year 3, a substantial proportion of plants exhibiting small early‐season size (or very delayed phenology) eventually reached relatively high biomass; these plants also tended to be the least damaged in that year (Figure 4b).

### 3.3 Reproduction

Very few plants flowered in the first and second year of the study. We therefore restricted our analysis of flowering to the last (third) full year of the common garden. In year 3, ~13% of plants produced flower buds, and of those, ~75% flowered. Similar to biomass, initial size and damage were the strongest predictors of whether a plant flowered in the last full year of the study, whereas a plant's source population history with biocontrol was not a significant predictor of its probability of bud formation or flowering. The timing of bud and flower formation was significantly impacted by herbivore damage in the first part of the growing season: heavier damage delayed the formation of both flower buds and flowers. We found no effect of population history on the timing of bud or flower formation (Table 2).

**Table 2:** Generalized linear mixed model of impact of common garden factors on the probability that a plant flowered in the third growing season, and on the timing of bud formation and flowering

Response Variable	Fixed Factors	*χ^2^*	*df*	*p*‐value
Probability of Flowering	Population History	0.588	2	.745
	Plot (Block)	8.12	3	.0435
	Early‐season Size	28.6	1	<.0001
	Defoliation	53.2	1	<.0001
Timing of bud formation (Julian date)	Population History	0.934	2	.627
	Plot (Block)	7.10	3	.0690
	Early‐season Size	0.225	1	.635
	Defoliation	15.8	1	<.0001
Timing of first flower (Julian date)	Population History	1.06	2	.588
	Plot (Block)	1.61	3	.656
	Early‐season Size	0.157	1	.692
	Defoliation	12.2	1	<.001

## 4 DISCUSSION

In a multi‐year common garden study, we found little evidence that a history of exposure has significantly altered an invasive plant's response to herbivory by its primary biocontrol agent. Irrespective of their population history of biocontrol*, L. salicaria* plants in our study suffered significant defoliation by *Neogalerucella* spp. beetles, and defoliation was an important predictor of plant performance, both within and across the years of the study. Overall, our common garden results revealed that herbivory by the biocontrol agent tended to magnify individual differences in plant performance—the most vigorous plants tended to escape herbivory, allowing them to grow larger more quickly, and eventually, to flower. Smaller, less vigorous individuals in our common garden suffered the highest rates of defoliation, ultimately reached a lower biomass, and were less likely to flower by the end of the study. Interestingly, this pattern of carry‐over across years revealed the only effect of a history with biocontrol that we found: plants from the original release populations exhibited a slightly steeper relationship between a plant's initial size and its season‐end biomass in the previous season, relative to plants from naïve populations. This finding could indicate that the introduction of biocontrol has selected for earlier spring phenology and/or increased allocation into growth prior to *Neogalerucella* spp. herbivory, which has implications for subsequent damage and plant fitness.

Overall, in spite of high variability in defoliation and biomass, plants in our study were surprisingly robust in terms of year‐to‐year survival. Indeed, some plants that seemed to have succumbed to herbivory early in our study had re‐emerged by later censuses and continued to persist until the final season. We conclude that, in the ~20 years since *Neogalerucella* spp. was first introduced for biocontrol in Ontario, any adaptation to its presence by *L. salicaria* has had little impact on plant defence strategies and biocontrol success in nature. Instead, the spatio‐temporal variability in ecological conditions that the plants encounter, especially herbivory, and plant phenotypic traits, such as size at the start of the season, were the main predictors of plant performance in the face of biocontrol. Our findings imply that the success of the *L. salicaria* biocontrol programme is not being significantly hampered by rapid adaptation to herbivory.

Our results contradict two prior studies of *L. salicaria* that reported findings consistent with rapid adaptation of anti‐herbivore defence. One, from our own laboratory, found evidence that, in a controlled greenhouse experiment using the same seed material, plants from populations with a history of biocontrol exhibited increased resistance and tolerance to *Neogalerucella* spp. herbivory compared to those from naïve populations (Stastny & Sargent, 2017). The second, performed as a common garden herbivory manipulation with populations from a separate (but similar) biocontrol programme in Minnesota, found that plants from populations with a history of biocontrol were marginally (*p* = .077) more tolerant of experimentally imposed herbivory than plants from naïve populations in the second, but not the first, year of the study (Quiram, 2013). Here, we report that in a multi‐year study in a natural setting, individual differences in plant growth and phenology tended to exceed among family and population differences, amplifying the variation in and consequences of herbivory across seasons. We suspect that a weak response to selection, which may be especially common in biocontrol settings (Holt & Hochberg, 1997), combined with a high degree of environmental variability in the common garden (both herbivory and environment were controlled in the greenhouse) were the key contributors to the lack of detectable effects of biocontrol history in the field. We expand on these ideas below.

In general, studies that explore plant adaption to population levels of herbivory are rare and tend to take place in controlled greenhouse settings (e.g. Sakata, Yamasaki, Isagi, & Ohgushi, 2014). Studies where plants are grown in more natural settings are much less common, with mixed support for local adaptation to herbivory. For example, Lehndal, Hamback, Ericson, and Agren, (2016), in a common garden study of nine populations from *L. salicaria's* native range that varied in the degree of herbivory by *Neogalerucella* spp., found that herbivore damage during the growing season, but not the level of herbivory measured at the source population, was the main predictor of a plant's flowering and seed output. In line with our findings, the authors concluded that the intensity of herbivory in the common garden, rather than local adaptation to the population's historic herbivory level, was the main driver of *L. salicaria's* flowering and seed output. These results contrasted with those of an earlier study by the same group (Lehndal & Ågren, 2015), which were more consistent with local adaptation to herbivory. The authors attributed their conflicting findings to differences in the selection regime across populations and space in the former study, which could have weakened net selection on anti‐herbivore defence.

Holt and Hochberg, (1997) outlined several explanations as to why postrelease adaptation of a target species may be weak in the context of biocontrol. First, the invasive species may lack genetic variation for defence traits. This scenario seems unlikely for *L. salicaria*, which exhibits genetic variation across a broad range of traits in its introduced range (R. Colautti, *pers. comm*.), including vigour, herbivore tolerance (Quiram, 2013), reproductive and vegetative size, and flowering phenology (Colautti & Barrett, 2011). Another possibility, that selection on defence is weak, seems unlikely given the clear impacts of herbivory on biomass and flowering revealed by our study. A more likely explanation for our findings is that fluctuating or variable selection for plant defence across populations, which is common in nature (Agrawal, 2011), weakens net selection on these traits (Cullen, Proost, & Volenberg, 2008; Muola et al., 2010). Many studies have demonstrated that herbivore damage in both the native and introduced ranges of *L. salicaria* varies spatially and temporally (Boag & Eckert, 2013; Denoth & Myers, 2005; Hovick & Carson, 2015; Lehndal et al., 2016; Quiram, 2013; St. Louis et al., 2020). Given sufficient gene flow, variable selection within and among populations could explain the lack of evidence for population divergence in our study. Future studies should examine the degree to which the underlying genetic covariance structure, gene flow, variable intra‐ and inter‐population selection, and phenotypic plasticity contribute to the lack of population divergence in response to biocontrol observed in our field study.

Our study offers several important insights into the trajectory and impacts of the *Neogalerucella* spp. biocontrol programme on the management of *L. salicaria* that were not previously evident due to a lack of large‐scale, long‐term studies under natural conditions. First, biocontrol has a strong impact on plant performance: defoliation by the biocontrol was an important predictor of annual fluctuations in plant biomass, and plants in our common garden took years to flower, compared to plants from the same populations grown in the greenhouse, which generally flowered within 3–4 months (M. Stastny, pers. obs.). This finding echoes other reports of the impacts of *Neogalerucella* spp. feeding on *L. salicaria* growth and phenology in field observations (e.g. Schat & Blossey, 2005). Yet, in spite of the impacts of herbivory on plant performance, we also observed an impressive ability of *L. salicaria* to tolerate biocontrol and persist, often with marked seasonal fluctuations in growth patterns. For example, it was not unusual for a plant's above‐ground portion to go missing for an entire growing season, only to reappear the following year. In these cases, the plant was clearly maintaining below‐ground biomass following a severe bout of herbivory, but at the expense of stem, leaf and flower production. In a study of impacts of herbivory on carbon storage in the short lived perennial *Rorippa palustris*, Sosnova and Klimesova, (2009) demonstrated that the timing of herbivory predicted whether plants would allocate to annual reproduction or reserve storage for regrowth the following spring, which could also explain why some of our plants disappeared temporarily. Overall, our study highlights what is impossible to establish from prerelease studies of biocontrol—that under heavy biocontrol pressure in a natural setting, plants sourced from a range of populations are heavily impacted by herbivory, and that this has long‐term, year‐over‐year impacts on fitness‐associated traits.

### 4.1 Conservation implications and management recommendations

Even in the noisy field environment, the impacts of the biocontrol agent on our study plants were clear: herbivory tended to result in a lower plant size at the end of a growing season, a lower likelihood of flowering/fruiting and multi‐season delays in the timing of flowering. These findings reinforce the empirical basis for the *Neogalerucella* spp. biocontrol programme, and parallel the interactions observed between these two species in their native range (Lehndal et al., 2016). Our study has important implications for one of the major concerns surrounding pest management—whether the target organism will evolve resistance to the control, lowering its efficacy (Szucs, Vercken, Bitume, & Hufbauer, 2019). We demonstrate that, in spite of the differences in tolerance and resistance evident under controlled experimental conditions (Quiram, 2013; Stastny & Sargent, 2017), in a more natural setting, plants from populations with a history of biocontrol exhibited few detectable differences in their ability to resist or tolerate herbivory. This finding suggests that, at least in the ~20 years since its inception, the success of the *L. salicaria* biocontrol programme is not being hampered by the evolution of increased defence in the invasive target species.

On the other hand, our study also demonstrates the striking ability of individual *L. salicaria* plants to persist in the face of strong herbivory, in spite of the marked spatio‐temporal variation in defoliation and its impacts on plant performance and allocation decisions. Variation in a plant's microsite and/or early‐season growth either enhanced or diminished its ability to grow large and eventually flower, magnifying growth and reproductive differences among individuals and their trajectories over time. Herbivory by *Neogalerucella* spp. in the field is known to be patchy (Denoth & Myers, 2005), suggesting that this spatial–temporal variability is a feature of the biocontrol agent's biology. Given this patchiness, and the robustness of *L. salicaria*, biocontrol on its own may provide insufficient pressure to adequately suppress growth or flowering in many populations, a finding supported by other studies (Denoth & Myers, 2005; Grevstad, 2006; Hovick & Carson, 2015; St. Louis et al., 2020). Therefore, managers should consider applying multiple control methods simultaneously, such as annual mowing or thinning of plants that have escaped defoliation, in order to support biocontrol programmes. This tactic could also reduce the possibility that certain locations become refuges for reproductive plants that escape herbivory year after year and help to maintain propagule pressure.

A final management implication of our study is that additional information and material should be collected from target populations prior to the release of biocontrol agents in order to enhance our ability to measure programme success and/or perform follow‐up studies. For example, in the present study, if seed material from a set of existing *L. salicaria* populations (including those where biocontrol was planned) had been gathered prior to the release of *Neogalerucella* spp., we would have been able to more directly search for evidence of a rapid evolutionary response. Moreover, metrics describing the prerelease target populations (e.g. plant density, height, flowering time, reproductive traits and plant community diversity) would provide future biologists an invaluable dataset from which we could determine key factors connected to biocontrol success, including its effect size, currently unknown for most release programmes (e.g. Grevstad, 2006; Hovick & Carson, 2015).

## Supporting information

Figure S1

Figure S2

Figure S3

Figure S4
