# Supplementary figures and images for: No evidence that rapid adaptation impedes biological control of an invasive plant
Source: Evol Appl. 2020 Aug 18;13(9):2472–83. doi: 10.1111/eva.13053 (PMC7513728; doi:10.1111/eva.13053)

## Slide 1
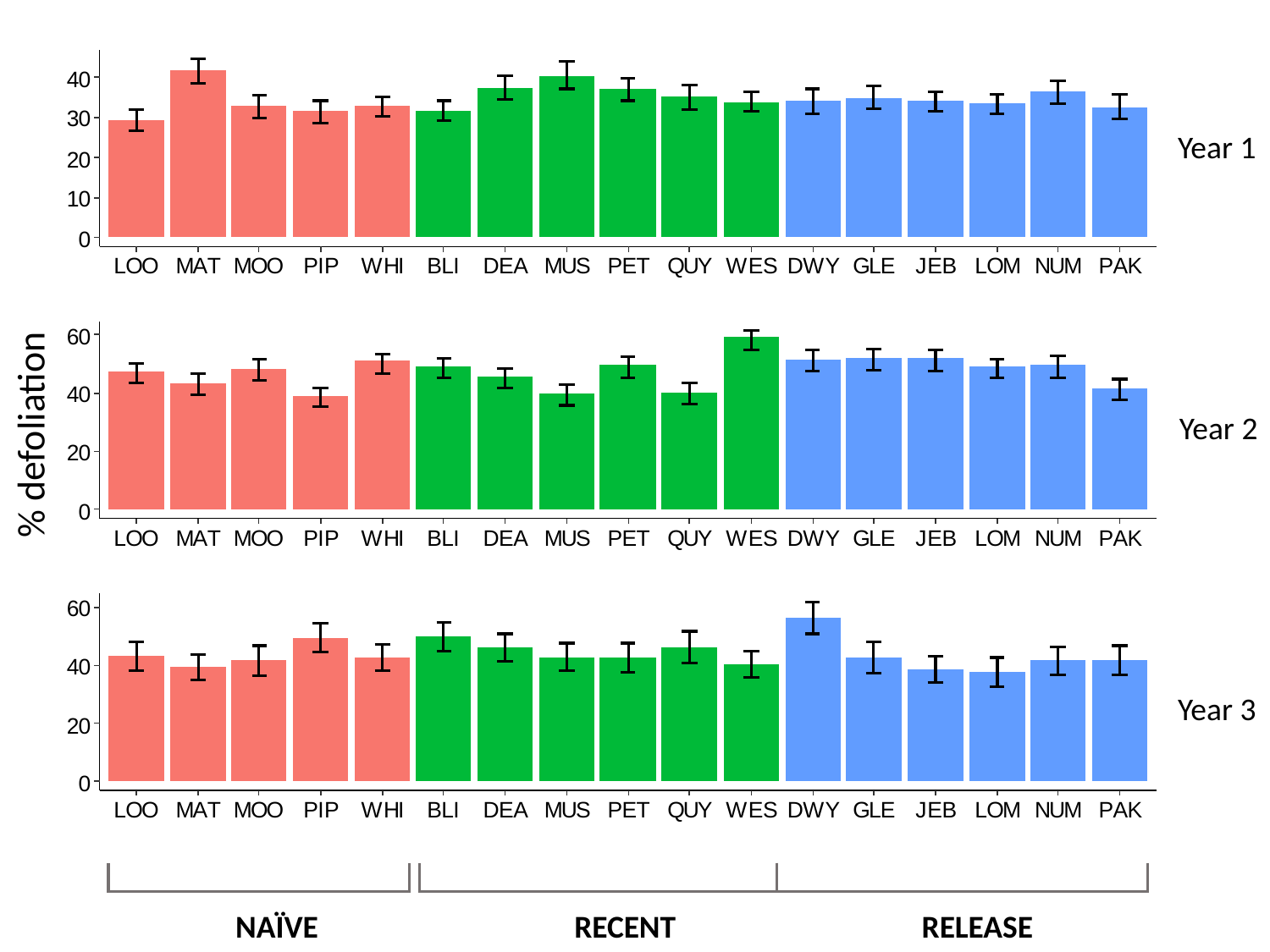

Year 1
Year 2
% defoliation
Year 3
NAÏVE
RECENT
RELEASE

Supplement: Supplementary file 1 — Figure S1 [file EVA-13-2472-s001.pptx]

## Slide 1
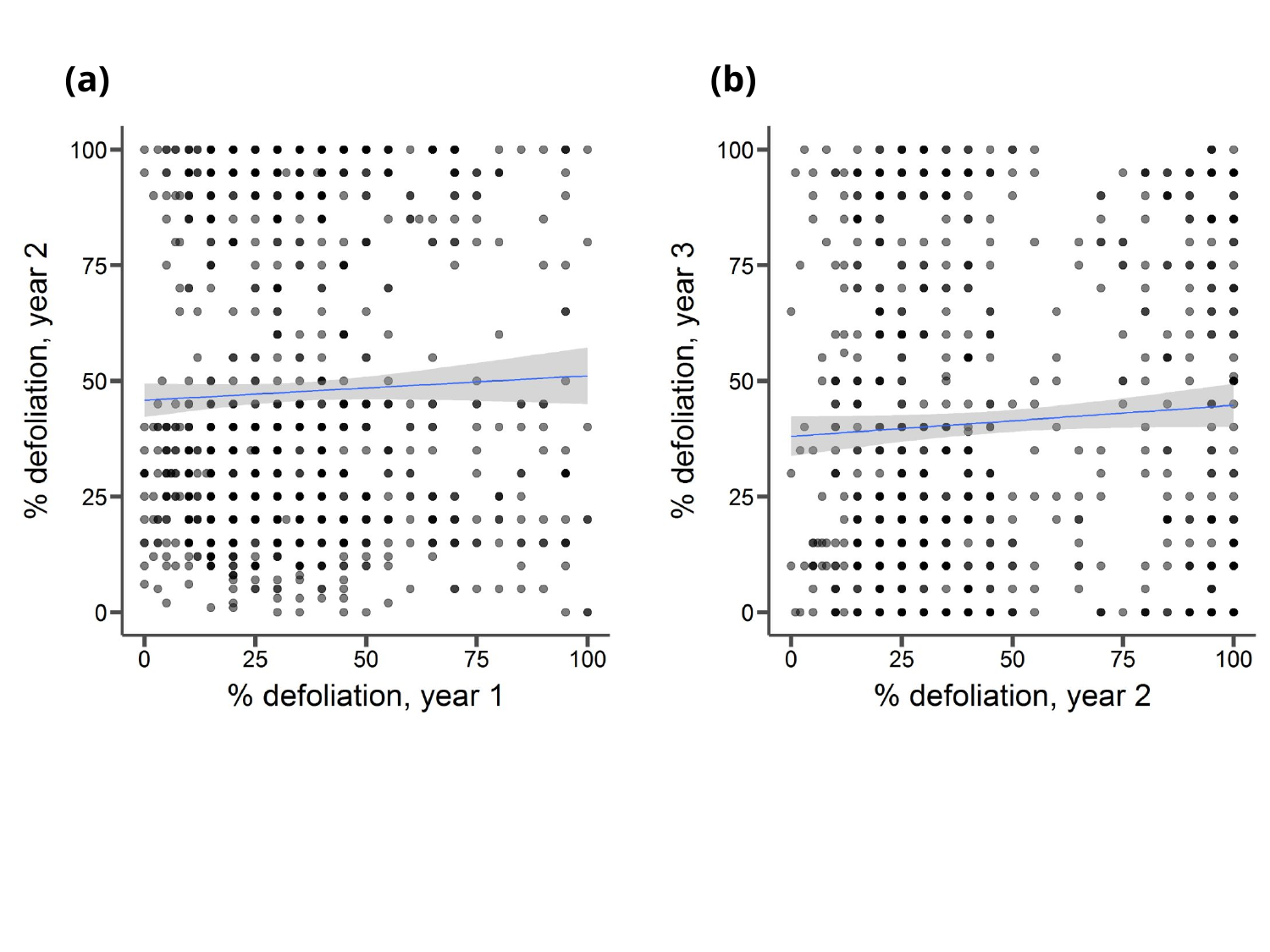

(a)
(b)

Supplement: Supplementary file 2 — Figure S2 [file EVA-13-2472-s002.pptx]

## Slide 1
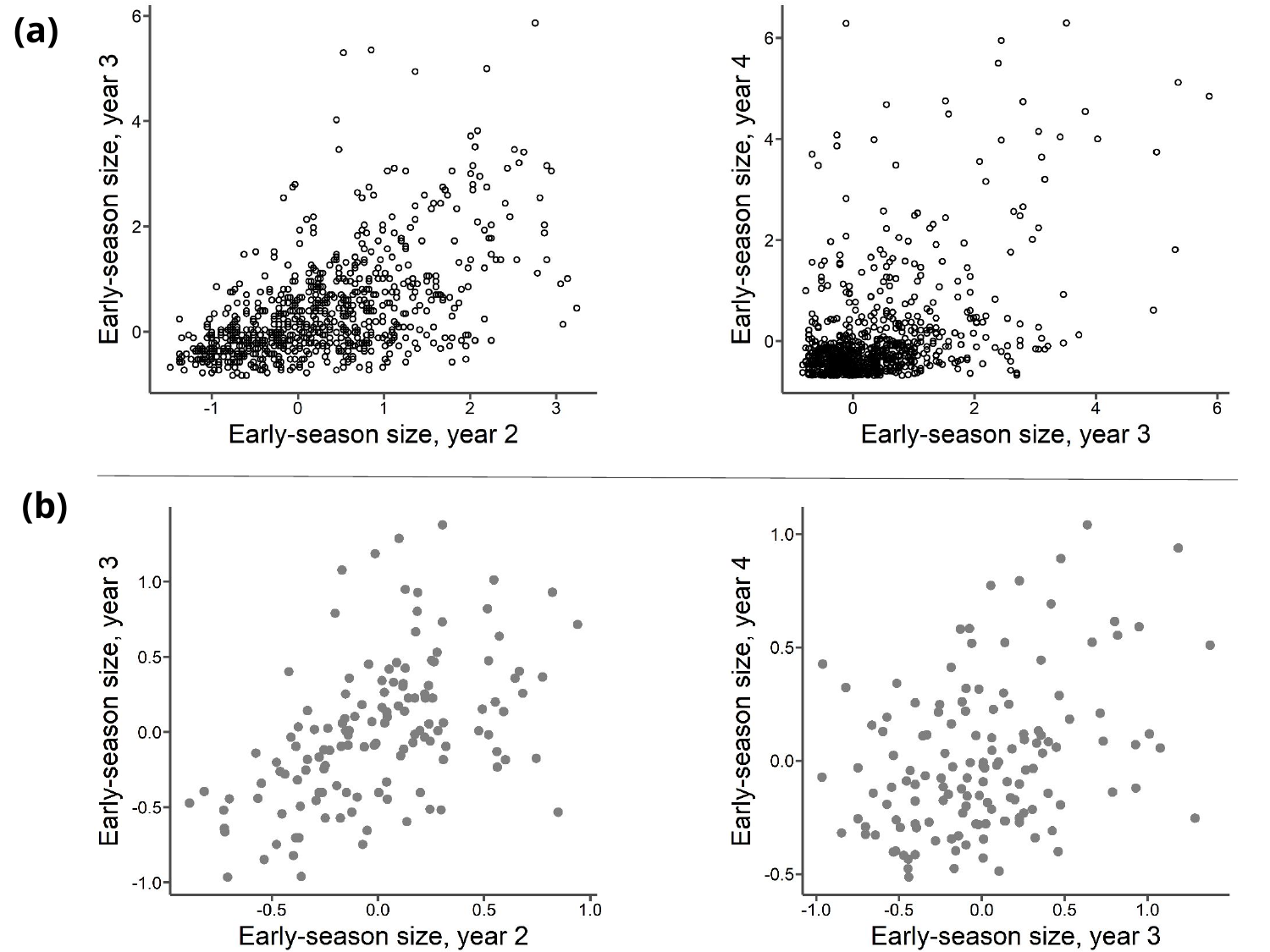

(a)
(b)

Supplement: Supplementary file 4 — Figure S4 [file EVA-13-2472-s004.pptx]
