# Supplementary material for: No evidence that rapid adaptation impedes biological control of an invasive plant
Source: Evol Appl. 2020 Aug 18;13(9):2472–83. doi: 10.1111/eva.13053 (PMC7513728; doi:10.1111/eva.13053)
Supplement: Supplementary file 3 — Figure S3 [file EVA-13-2472-s003.pptx]

## Slide 1
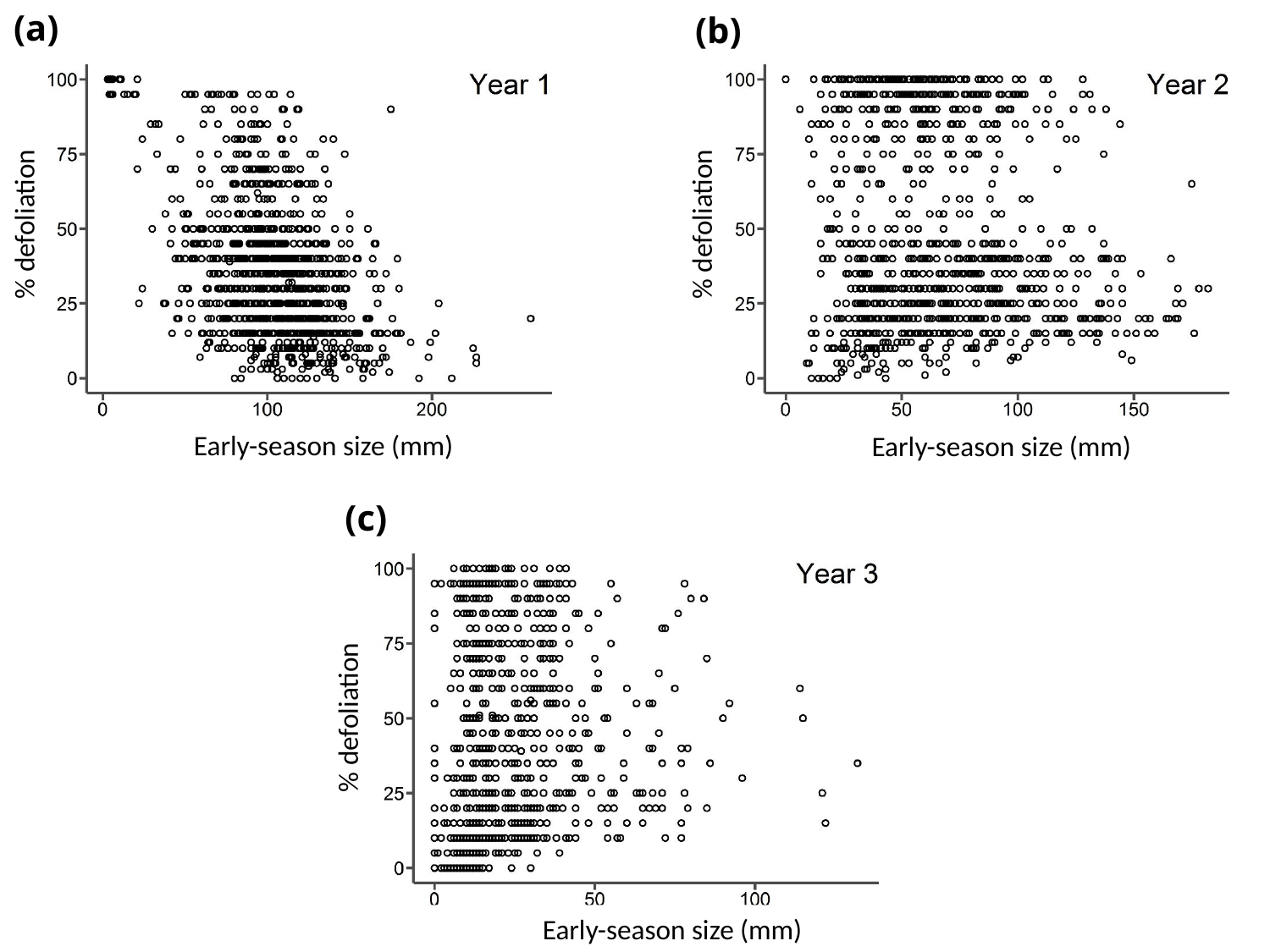

(a)
(b)
% defoliation
% defoliation
Early-season size (mm)
Early-season size (mm)
(c)
% defoliation
Early-season size (mm)
